# Moderating effects of general self-efficacy on courtesy stigma and anxiety and depressive symptoms of parents of children with autism spectrum disorder

**DOI:** 10.3389/fpsyt.2024.1454004

**Published:** 2024-10-03

**Authors:** Jiao Tong, Xu Chen, Bin Wang, Tao Wang, Xue Wang, Shan Ma, Dongdong Shi, Xin Wang, Dongmei Yan

**Affiliations:** ^1^ Lianyungang Maternal and Child Health Hospital Affiliated to Kangda College of Nanjing Medical University, Lianyungang, China; ^2^ The First Affiliated Hospital of Dalian Medical University, Dalian, China

**Keywords:** moderating effects, courtesy stigma, general self-efficacy, anxiety symptoms, depressive symptoms, parents, ASD

## Abstract

**Background:**

Stigma, anxiety and depressive symptoms are highly prevalent in parents of children with autism spectrum disorder (ASD) and may have a detrimental impact on the rehabilitation and treatment of children with ASD, ultimately leading to more behavioral issues and higher rates of disability. Therefore, the purpose of this study was to identify the association between general self-efficacy, courtesy stigma, and anxiety and depressive symptoms, and to further discuss whether general self-efficacy moderated the association between courtesy stigma and anxiety and depressive symptoms in parents of children with ASD.

**Methods:**

A total of 409 parents of children with ASD from Lianyungang, Jiangsu Province, Eastern China participated in a cross-sectional survey. A structured questionnaire was used to collect sociodemographic characteristics, courtesy stigma, general self-efficacy, anxiety symptoms, and depressive symptoms. Hierarchical multiple regression was used to assess the associations of courtesy stigma, general self-efficacy and courtesy stigma × general self-efficacy interaction with anxiety and depressive symptoms. Simple slope analysis was used to visualize the interaction.

**Results:**

The courtesy stigma of parents of children with ASD was positively correlated with anxiety (*B* = 0.374, *P* < 0.001) and depressive symptoms (*B* = 0.366, *P* < 0.001). General self-efficacy was negatively correlated with anxiety (*B* = -0.200, *P* < 0.001) and depressive symptoms (*B* = -0.210, *P* < 0.001). The association between courtesy stigma and anxiety symptoms was different in the high (1 standard deviation (SD) above the mean, *b* = 0.258, standard error (SE) = 0.056, *t* = 4.567, *P* < 0.001) and low (1 SD below the mean, *b* = 0.470, SE = 0.053, *t* = 8.870, *P* < 0.001) groups of general self-efficacy. In addition, the association between courtesy stigma and depressive symptoms was also different in the high (1 SD above the mean, *b* = 0.241, SE = 0.056, *t* = 4.268, *P* < 0.001) and low (1 SD below the mean, *b* = 0.469, SE = 0.053, *t* = 8.844, *P* < 0.001) groups of general self-efficacy.

**Conclusions:**

General self-efficacy could moderate the impact of courtesy stigma on anxiety and depressive symptoms. Therefore, among parents of children with ASD who experienced high courtesy stigma, enhancing general self-efficacy could be an effective strategy to reduce anxiety and depressive symptoms in this population.

## Introduction

Autism spectrum disorder (ASD) is a complex neurodevelopmental disorder characterized by challenges in social interaction, communication deficit, and restricted and repetitive behavioral patterns or interests, typically identified in early life (1). According to the Autism and Developmental Disabilities Monitoring (ADDM) Network of the Centers for Disease Control and Prevention (CDC), approximately 1 in 36 8-year-old children are diagnosed with ASD (2), which has become one of the most common neurodevelopmental disorders in the world (3). According to statistics, the prevalence of ASD among children aged 6-12 in China is about 0.70% (95% *CI*: 0.64%–0.74%) (4). In China, although government has implemented several supportive polices for ASD including clinical diagnosis and treatment, early screening and rehabilitation guidance, the challenges and prognosis faced by these children are still influenced by multiple factors. This not only includes the basic clinical characteristics, but also the family nurturing environment and the psychological characteristics of parents, which significantly affect the progress of the disorder treatment (5). On a global scale, the psychological well-being of parents of children with ASD has emerged as a significant barrier impacting the development of the condition and the effectiveness of rehabilitation training for these children (6).

Parents of children with ASD may be more likely to suffer from psychological disorders than parents of typically developing children (7–9). A meta-analysis study revealed that the global median prevalence of parental anxiety and depressive symptoms in children with ASD were approximately 33% (95% *CI*: 20-48%) and 31% (95% *CI*: 24-38%), respectively (10). However, screening and treatment for anxiety and depressive symptoms in parents of children with ASD are often neglected, and few parents have access to or actively seek the required health care services (11). This may have a detrimental impact on the anxiety and depressive symptoms levels of parents. Parents with psychological disorders may impede compliance and effectiveness for children diagnosed with ASD during early intervention (12). Missing the optimal intervention timing not only compromises rehabilitation outcomes, but also disrupts the family nurturing environment, exacerbating behavioral problems in children with ASD (13–15). Anxiety and depressive symptoms in parents of children with ASD may serve as potential barriers to timely and effective provision of rehabilitation therapy, thereby exacerbating the economic and social burden associated with ASD care, ultimately jeopardizing the overall treatment outcomes for the affected children (16, 17). However, anxiety and depressive symptoms can be treated through appropriate intervention measures. Empirical research has provided evidence that psychological intervention targeting primary caregivers of children with ASD significantly improve emotional development and parenting skills, thereby exerting a positive influence on the well-being of children with ASD (18). Given the prevalence, detrimental effects, and treatability of anxiety and depressive symptoms in parents of children with ASD, it is crucial to further investigate the associated factors and underlying mechanism. This will help identify specific characteristics of individuals who require attention and develop effective intervention measures.

In recent years, an increasing number of studies have focused on the stigma experienced by parents of children with ASD. The concept of “stigma” was first introduced by Goffman, and he described stigma as the attribute(s) that one possesses which discredits an individual in a specific context and spoils one’s identity through its relationships with stereotypes (19). Courtesy stigma refers to the general public’s negative beliefs, attitudes, and behaviors toward the associates of people with discredited characteristics (20). Due to a lack of public knowledge of ASD, many affected children often encounter stereotypes, courtesy stigma, misconceptions, and discrimination from mainstream society (21). Courtesy stigma not only impact on children with ASD but also significantly affect their loved ones (15). There is a widespread feeling of stigma surrounding ASD within communities, with up to 95% of American households reporting experiencing some form of societal disapproval (22). In some countries, such as Vietnam, ASD has been variously conceptualized as a disease, a “family problem,” and karmic demerit (23). Somalian families in the United Kingdom and members of the Aboriginal and Torres Strait Island community in Australia with autistic family members also reported high levels of stigma toward their autistic children (24–26). The findings from in-depth interview indicated that parents of children diagnosed with ASD, particularly mothers, encountered a profound sense of felt and enacted stigma in public settings, specially within educational and community environments (27). However, the stigmatization of collectivist culture in China is more prominent than in other countries, and individuals who deviate from social norms often experience a greater sense of stigma. In China, there is a greater emphasis on social acceptance and validation. When a child with a disability fails to meet societal expectations, parents often exhibit heightened sensitivity to the negative perceptions of the public (28). This may have an important negative effect on the primary caregivers of children with ASD, exacerbating their anxiety, depression, and other adverse psychological traits (29). Research has also found that a positive correlation exists between stigma and psychological distress in parents of children diagnosed with ASD (30). Thus, courtesy stigma may play an important role in the development of anxiety and depressive symptoms in parents of children with ASD in eastern China.

Self-efficacy is a concept in social cognitive theory proposed by Bandura, which primarily refers to an individual’s belief in their ability to perform a specific behavior or produce a desired outcome through certain actions (31). Research has shown mothers of children with ASD exhibiting lower level of self-efficacy (32), and a negative correlation between parental self-efficacy and psychopathology (33). It is worth noting that in other population, such as pregnant women, individuals with low self-efficacy may experience elevated levels of anxiety and depressive symptoms (34). Self-efficacy plays a crucial role in ameliorating postoperative negative emotions among prostate cancer patients, including anxiety and depressive symptoms (35). During the COVID-19 pandemic, self-efficacy has also played a positive role in keeping the public optimistic and mentally healthy (36). However, limited research has been conducted on how self-efficacy affect anxiety and depressive symptoms in parents of children with ASD. In addition, in recent years, an increasing number of research has focused on the significant moderating effect of self-efficacy (37–39). Empirical studies have demonstrated that self-efficacy can moderate the relationship between stigma and emotional reaction (40). However, few studies have explored whether general self-efficacy moderates the association between courtesy stigma and anxiety and depressive symptoms in parents of children with ASD. Therefore, we speculate that general self-efficacy not only directly impact anxiety and depressive symptoms but also might be a positive resource in buffering the association between courtesy stigma and anxiety and depressive symptoms in parents of children with ASD.

**Figure 1:**
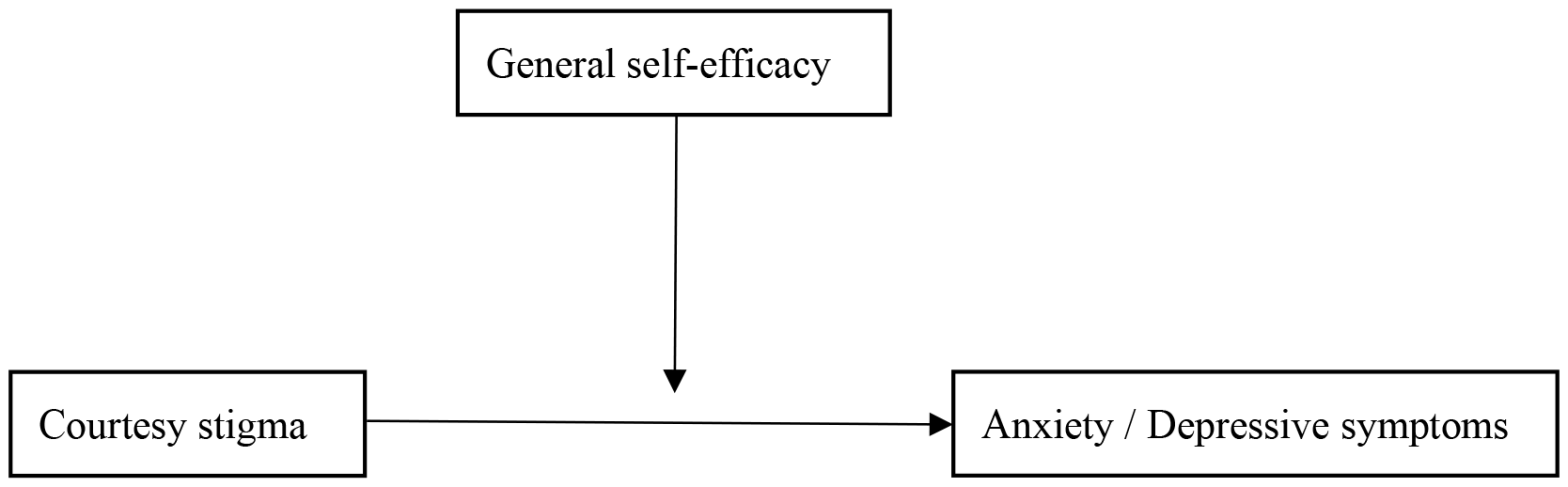
Hypothesized model.

In general, there have been many studies on the impact of courtesy stigma on anxiety and depressive symptoms, but few have explored the role of general self-efficacy in the relationship between courtesy stigma and anxiety and depressive symptoms. In addition, to the best of our knowledge, there is currently lack of research exploring the association between courtesy stigma, general self-efficacy and anxiety and depressive symptoms in parents of children with ASD in eastern China. Considering this issue, we have conducted a cross-sectional study in Lianyungang, Jiangsu Province, Eastern China to validate the following three hypotheses in parents of children with ASD (**Figure 1**).

Hypothesis 1 Courtesy stigma is positively related to anxiety and depressive symptoms.
Hypothesis 2 General self-efficacy is negatively related to anxiety and depressive symptoms.
Hypothesis 3 The association between courtesy stigma and anxiety and depressive symptoms could be moderated by general self-efficacy.

## Materials and methods

### Study design and participants

A cross-sectional survey was conducted in the rehabilitation department of a large specialized hospital and 10 rehabilitation centers for children with special needs in Lianyungang, Jiangsu Province, Eastern China from October 2022 to February 2023. Systematic random sampling technique was used to select participants. Participants were the parent who claimed to be living with and providing the most care services to the child with ASD at the study site. One parent of each child with ASD was invited to participate in this study and fill out a structured questionnaire. The inclusion criteria for participants were: (1) raising a child with ASD diagnosed by an occupational clinician; (2) age was greater than or equal to 18 years; (3) raising children with ASD who were less than or equal to 12 years and had no other serious diseases; (4) without any mental illness and able to understand the questionnaire; (5) consent to participate in the study. The investigators were a fixed survey team of five people who were trained and familiar with the content of the questionnaire. The training content mainly included the purpose of the study, the screening of the research object, the general requirements of the survey and the matters needing attention. Before inviting participants to participate in the study, the investigators clearly explained the purpose of the study, the process of the study, the rights of the participants, and the confidentiality of the data, etc., and informed consent was obtained from the participants. The investigators distributed the questionnaires and were responsible for guidance and interpretation, and all questionnaires were recovered on the spot. A total of 430 parents of children with ASD were recruited in this study, of which 21 parents who did not complete the questionnaire due to time issues were excluded. Finally, this study included 409 parents of children with ASD, the participation rate is 95.1%. As a token of appreciation for participation, each parent who completed the questionnaire received a small toy.

### Measures

#### Sociodemographic characteristics questionnaire

The research team developed the sociodemographic characteristics questionnaire based on literature reading and consultation with experts in relevant fields. It consisted of 8 questions to collect information on the sociodemographic characteristics of the participants and their children with ASD, including the children’s age, sex, ASD Severity, parents’ age, sex, residence, unemployment (not having a stable job at the moment), and educational status.

#### The Perceived Courtesy Stigma Scale (PCSS)

The Perceived Courtesy Stigma Scale (PCSS) was used to measure the courtesy stigma of parents of children with ASD (30). It consisted of 7 items, each rated on a 4-point Likert scale ranging from 0 (strongly disagree) to 3 (strongly agree). One sample item was “Most people do blame parents for the ASD of their children”. Each item score was summed to obtain a total score. A higher total score indicated a higher level of courtesy stigma. The scale has shown good internal consistency in studies of parents of children with ASD in many countries, including China (20, 41). In the present study, Cronbach’s alpha was 0.893.

#### The General Self-Efficacy Scale (GSES)

The General Self-Efficacy Scale (GSES) was used to assess the general self-efficacy of parents of children with ASD (42). It consisted of 10 items, each rated on a 4-point Likert scale ranging from 1 (not at all true) to 4 (exactly true). One sample item was “If I try my best to do it, I can always solve the problem”. The total score was the sum of the scores for each item. The lower the total score, the lower the general self-efficacy level. The scale has been widely used in a variety of populations in China and has shown good reliability (43, 44). In the present study, Cronbach’s alpha was 0.907.

#### The Generalized Anxiety Disorder-7 (GAD-7)

The Generalized Anxiety Disorder-7 (GAD-7) was used to measure the anxiety symptoms of parents of children with ASD (45). It assessed subjective anxiety symptoms of parents of children with ASD over the past two weeks. It consisted of 7 items, each rated on a 4-point Likert scale ranging from 0 (not at all) to 3 (almost every day). Total score range was 0 to 21, with higher scores indicating more severe self-reported anxiety symptoms. Its effectiveness has been proved in China (46, 47). In the present study, Cronbach’s alpha was 0.934.

#### The Patient Health Questionnaire-9 (PHQ-9)

The Patient Health Questionnaire-9 (PHQ-9) was used to assess the depressive symptoms of parents of children with ASD (48). It was widely regarded as simple self-management tool to screen for depressive symptoms. It consisted of 9 items, each rated on a 4-point Likert scale ranging from 0 (not at all) to 3 (almost every day). Total scores range from 0 to 27, with higher scores indicating more severe self-reported depressive symptoms. It has been validated in mothers of children with ASD (32). In the present study, Cronbach’s alpha was 0.910.

### Statistical analysis

Complete and non-missing questionnaires were coded and entered into a database established by EpiData3.1 (EpiData Association, Odense, Denmark) software. After checked, export to SPSS21.0 (IBM Corporation, Armonk, State of New York) for statistical analysis. Descriptive statistical analysis was used to describe the sociodemographic characteristics of children with ASD and their parents, parental courtesy stigma, general self-efficacy, anxiety symptoms, and depressive symptoms. Continuous variables were described as mean and standard deviation (SD) or as median and interquartile rang. Categorical variables were described as frequency and percentage. Mann-Whitney U test and Kruskal-Wallis H test were used to examine the association of sociodemographic characteristics with anxiety and depressive symptoms in parents of children with ASD. Pearson and spearman correlation analyses were used to explore the correlation between continuous variables. Hierarchical multiple regression analysis was used to examine the relationships among courtesy stigma, general self-efficacy and anxiety and depressive symptoms, and to explore the moderating role of general self-efficacy on the association between courtesy stigma and anxiety and depressive symptoms. In step 1, sociodemographic characteristics as potential controlling variables were added. Courtesy stigma and general self-efficacy were added in step 2. In step 3, the product of courtesy stigma and general self-efficacy was added. All continuous variables were standardized before hierarchical multiple regression analysis to reduce the potential effects of multicollinearity (49). If the interaction effect was statistically significant, a simple slope analysis was performed following Aiken and West’s procedures to visualize the moderating effect of general self-efficacy (50). All statistical analysis were two-sided tests, and *P* value was less than 0.05 was considered statistically significant.

## Results

### Sociodemographic characteristics

A total of 409 parents of children with ASD were included in this study, with a mean age of 33.30 years (SD = 5.10; range = 21-53 years). More than three-fifths of the participants (63.8%) were female, and more than half (53.5%) were urban residents. Almost half of the participants (50.1%) were currently unemployed, and more than two-fifths (41.8%) had a college level or higher. Among children with ASD, the majority (66.7%) were younger than 6 years of age. More than two-thirds of the children (69.7%) were male, and almost one-fifth (19.1%) had severe current symptoms. In the current study, the median anxiety symptoms score was 3.00 (Interquartile rang = 1.00-7.00 scores) and the median depressive symptoms score was 5.00 (Interquartile rang = 1.00-9.00 scores). The univariate analyses indicated that there were significant differences in anxiety symptoms on two sociodemographic characteristics: the severity of ASD in children and parents’ unemployment status (*P* < 0.05). The univariate analyses indicated that there were significant differences in depressive symptoms on four sociodemographic characteristics: children’s ASD severity, parents’ residence, unemployment status, and educational status (*P* < 0.05) (**Table 1**).

**Table 1:** Sociodemographic characteristics of children and parents and univariate analysis of factors associated with anxiety and depressive symptoms.

Variables	n (%)	Anxiety symptoms		*P*	Depressive symptoms		*P*
		median	Interquartile rang		median	Interquartile rang	
Children							
Age				0.154			0.072
<6	273 (66.7)	3.00	1.00-7.00		4.00	1.00-9.00	
≥6	136 (33.3)	4.00	1.00-8.00		5.00	2.00-9.00	
Sex				0.450			0.657
Female	124 (30.3)	3.00	0.00-7.00		4.50	1.25-9.00	
Male	285 (69.7)	4.00	1.00-7.00		5.00	1.00-9.00	
ASD severity				**<0.001**			**<0.001**
Mild	122 (29.8)	2.00	0.00-5.25		3.00	0.00-7.00	
Moderate	209 (51.1)	4.00	1.00-7.00		5.00	2.00-9.00	
Severe	78 (19.1)	7.00	1.00-10.00		7.00	3.00-12.00	
Parents							
Age				0.436			0.172
≤30	127 (31.1)	3.00	1.00-7.00		4.00	0.00-8.00	
31-45	267 (65.3)	4.00	1.00-7.00		5.00	1.00-9.00	
>45	15 (3.7)	4.00	2.00-12.00		5.00	3.00-8.00	
Sex				0.058			0.145
Female	261 (63.8)	4.00	1.00-7.50		5.00	1.00-9.00	
Male	148 (36.2)	3.00	0.00-7.00		4.00	1.00-8.00	
Residence				0.116			**0.001**
Urban	219 (53.5)	3.00	0.00-7.00		4.00	1.00-7.00	
Rural	190 (46.5)	4.00	1.00-8.00		6.00	2.00-9.00	
Unemployment				**0.020**			**0.001**
Yes	205 (50.1)	4.00	1.00-9.00		5.00	2.00-10.00	
No	204 (49.9)	3.00	0.00-7.00		4.00	1.00-7.00	
Educational status				0.154			**0.028**
Junior high school or less	131 (32.0)	4.00	1.00-10.00		6.00	1.00-12.00	
High school	107 (26.2)	3.00	0.00-7.00		4.00	0.00-8.00	
College or above	171 (41.8)	3.00	1.00-7.00		4.00	1.00-8.00	

ASD, autism spectrum disorder; All significant *P* are displayed in bold.

### Correlations of the main study variables

The correlations among age, courtesy stigma, general self-efficacy, anxiety symptoms, and depressive symptoms were presented in **Table 2**. Courtesy stigma was positively correlated with anxiety and depressive symptoms (*r* = 0.405 and *r* = 0.394, respectively, *P* < 0.01). General self-efficacy was negatively correlated with anxiety and depressive symptoms (*r* = -0.382 and *r* = -0.388, respectively, *P* < 0.01). In addition, courtesy stigma was negatively correlated with general self-efficacy (*r* = -0.279, *P* < 0.01), and anxiety symptoms was positively correlated with depressive symptoms (*r* = 0.801, *P* < 0.01).

**Table 2:** Descriptive statistics and inter-correlations of main study variables.

Variables	Median (*P*_25_, *P*_75_)	Mean ± SD	Correlations (*r*)					
			1	2	3	4	5	6
1. Age of children	4.00(3.00, 6.00)		1					
2. Age of parents	33.00(30.00, 36.00)		0.236**	1				
3. Courtesy stigma		7.48 ± 4.13	0.015	0.055	1			
4. General self-efficacy		26.35 ± 4.43	-0.121*	-0.045	-0.279**^, a^	1		
5. Anxiety symptoms	3.00(1.00, 7.00)		0.103*	-0.013	0.405**	-0.382**	1	
6. Depressive symptoms	5.00(1.00, 9.00)		0.117*	0.049	0.394**	-0.388**	0.801**	1

SD, standard deviations; * *P* < 0.05, ** *P* < 0.01 (two-tailed); ^a^ Pearson correlation analysis.

### Moderation of the relationship between courtesy stigma and anxiety and depressive symptoms

The results of hierarchical multiple regression analysis are displayed in **Tables 3**, **4**. First, the linear combination of sociodemographic characteristics controlling variables (children’s age, sex, ASD severity, parents’ age, sex, residence, unemployment, educational status) significantly explained anxiety symptoms (*F* = 4.229, *R* ^2^ = 0.096, *P* < 0.001) and depressive symptoms (*F* = 4.897, *R* ^2^ = 0.110, *P* < 0.001).

**Table 3:** Hierarchical multiple regression results of anxiety symptoms.

Variables	Step 1				Step 2				Step 3			
	*B*	Beta	95% *CI* for *B*	*P*	*B*	Beta	95% *CI* for *B*	*P*	*B*	Beta	95% *CI* for *B*	*P*
Children												
Age	0.075	0.075	-0.025-0.174	0.140	0.056	0.056	-0.032-0.144	0.211	0.046	0.046	-0.041-0.133	0.298
Sex (Ref: Male)												
Female	-0.077	-0.036	-0.283-0.128	0.459	-0.080	-0.037	-0.261-0.100	0.383	-0.088	-0.041	-0.267-0.090	0.332
ASD severity (Ref: Mild)												
Moderate	0.292	0.146	0.073-0.511	**0.009**	0.177	0.089	-0.017-0.371	0.073	0.161	0.081	-0.031-0.353	0.100
Severe	0.627	0.247	0.345-0.909	**<0.001**	0.434	0.171	0.184-0.684	**0.001**	0.397	0.156	0.149-0.645	**0.002**
Parents												
Age	-0.020	-0.020	-0.120-0.080	0.694	-0.038	-0.038	-0.126-0.049	0.390	-0.035	-0.035	-0.122-0.051	0.424
Sex (Ref: Male)												
Female	0.196	0.094	-0.016-0.409	0.070	0.133	0.064	-0.054-0.320	0.162	0.105	0.050	-0.081-0.290	0.268
Residence (Ref: Urban)												
Rural	0.083	0.042	-0.126-0.293	0.435	0.066	0.033	-0.119-0.250	0.484	0.072	0.036	-0.111-0.254	0.441
Unemployment (Ref: No)												
Yes	0.190	0.095	-0.025-0.405	0.083	0.190	0.095	-0.001-0.382	0.051	0.192	0.096	0.003-0.381	**0.047**
Educational status (Ref: Junior high school or less)												
High school	-0.180	-0.079	-0.439-0.079	0.173	0.007	0.003	-0.223-0.237	0.953	0.002	0.001	-0.225-0.230	0.985
College or above	-0.085	-0.042	-0.341-0.171	0.513	-0.035	-0.017	-0.261-0.191	0.759	-0.031	-0.015	-0.255-0.192	0.784
Courtesy stigma					0.374	0.374	0.286-0.462	**<0.001**	0.364	0.364	0.277-0.451	**<0.001**
General self-efficacy					-0.200	-0.200	-0.290--0.111	**<0.001**	-0.193	-0.193	-0.281--0.104	**<0.001**
Courtesy stigma × General self-efficacy									-0.106	-0.139	-0.169--0.043	**0.001**
*F*	4.229			**<0.001**	14.478			**<0.001**	14.533			**<0.001**
*R* ^2^	0.096				0.305				0.324			
Δ*R* ^2^	0.096				0.209				0.019			

ASD, autism spectrum disorder; B, unstandardized coefficients; Beta, standardized coefficients; CI, confidence interval; All significant *P* are displayed in bold.

**Table 4:** Hierarchical multiple regression results of depressive symptoms.

Variables	Step 1				Step 2				Step 3			
	*B*	Beta	95% *CI* for *B*	*P*	*B*	Beta	95% *CI* for *B*	*P*	*B*	Beta	95% *CI* for *B*	*P*
Children												
Age	0.087	0.087	-0.012-0.186	0.083	0.068	0.068	-0.019-0.155	0.127	0.057	0.057	-0.029-0.143	0.192
Sex (Ref: Male)												
Female	-0.001	0.000	-0.204-0.203	0.995	-0.003	-0.001	-0.182-0.176	0.972	-0.012	-0.005	-0.188-0.165	0.895
ASD severity (Ref: Mild)												
Moderate	0.254	0.127	0.037-0.472	**0.022**	0.141	0.071	-0.051-0.333	0.149	0.124	0.062	-0.065-0.314	0.199
Severe	0.556	0.219	0.276-0.835	**<0.001**	0.364	0.143	0.116-0.612	**0.004**	0.324	0.127	0.078-0.569	**0.010**
Parents												
Age	-0.006	-0.006	-0.104-0.093	0.911	-0.024	-0.024	-0.111-0.063	0.584	-0.021	-0.021	-0.106-0.065	0.632
Sex (Ref: Male)												
Female	0.145	0.070	-0.065-0.356	0.176	0.083	0.040	-0.103-0.268	0.381	0.052	0.025	-0.132-0.235	0.580
Residence (Ref: Urban)												
Rural	0.191	0.095	-0.017-0.399	0.072	0.174	0.087	-0.009-0.356	0.063	0.180	0.090	0.000-0.360	0.050
Unemployment (Ref: No)												
Yes	0.246	0.123	0.033-0.459	**0.024**	0.242	0.121	0.053-0.432	**0.012**	0.244	0.122	0.057-0.430	**0.011**
Educational status (Ref: Junior high school or less)												
High school	-0.181	-0.080	-0.438-0.076	0.167	0.006	0.003	-0.222-0.234	0.959	0.001	0.000	-0.224-0.226	0.994
College or above	-0.118	-0.058	-0.372-0.136	0.362	-0.065	-0.032	-0.289-0.159	0.566	-0.061	-0.030	-0.282-0.160	0.587
Courtesy stigma					0.366	0.366	0.279-0.453	**<0.001**	0.355	0.355	0.269-0.441	**<0.001**
General self-efficacy					-0.210	-0.210	-0.298--0.122	**<0.001**	-0.202	-0.202	-0.289--0.114	**<0.001**
Courtesy stigma × General self-efficacy									-0.114	-0.150	-0.176--0.052	**<0.001**
*F*	4.897			**<0.001**	15.335			**<0.001**	15.573			**<0.001**
*R* ^2^	0.110				0.317				0.339			
Δ*R* ^2^	0.110				0.208				0.022			

ASD, autism spectrum disorder; B, unstandardized coefficients; Beta, standardized coefficients; CI, confidence interval; All significant *P* are displayed in bold.

**Figure 2:**
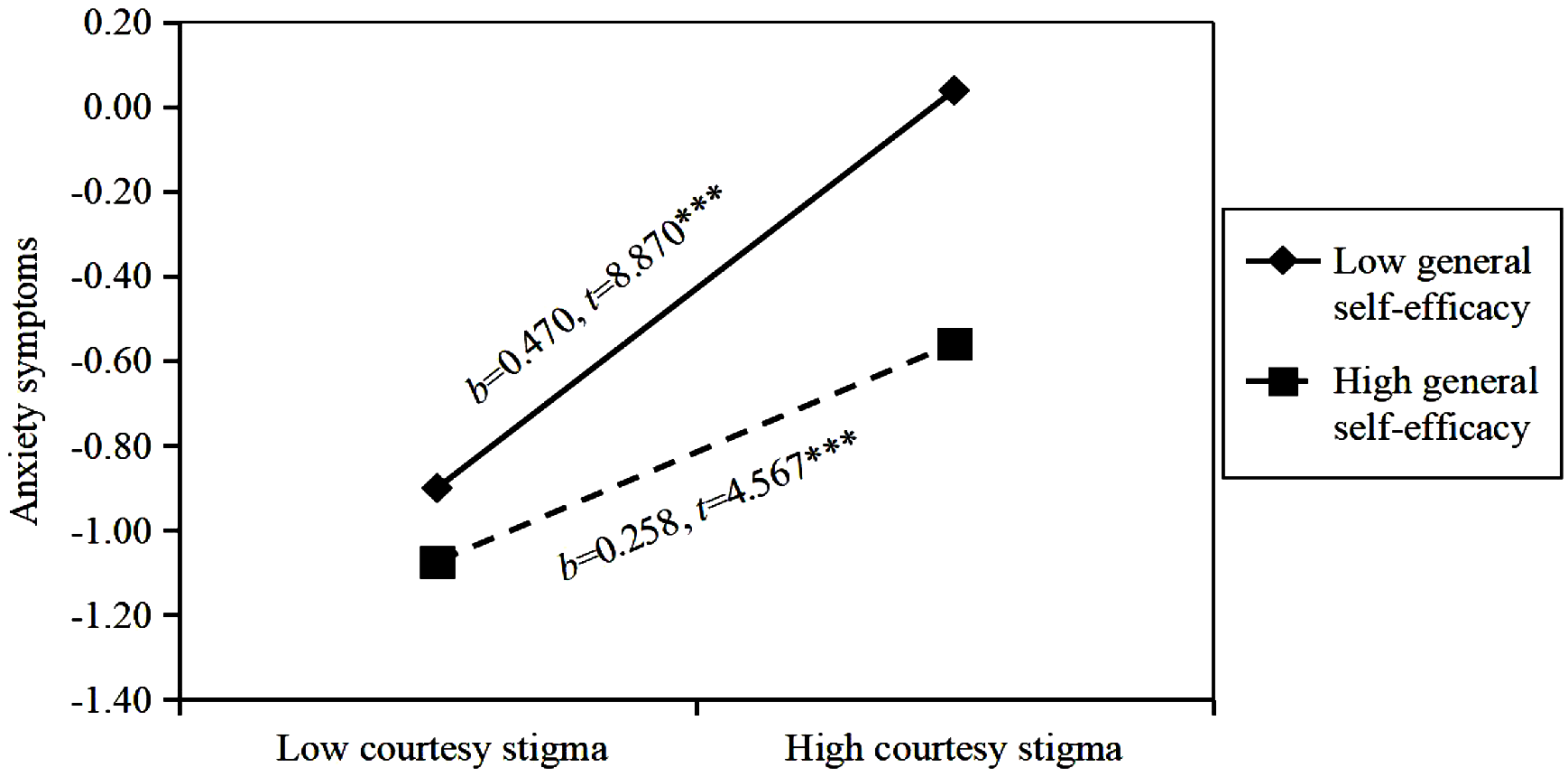
Moderating effect of general self-efficacy on the relationship between courtesy stigma and anxiety symptoms. *b* = slope; *** *P*<0.001.

**Figure 3:**
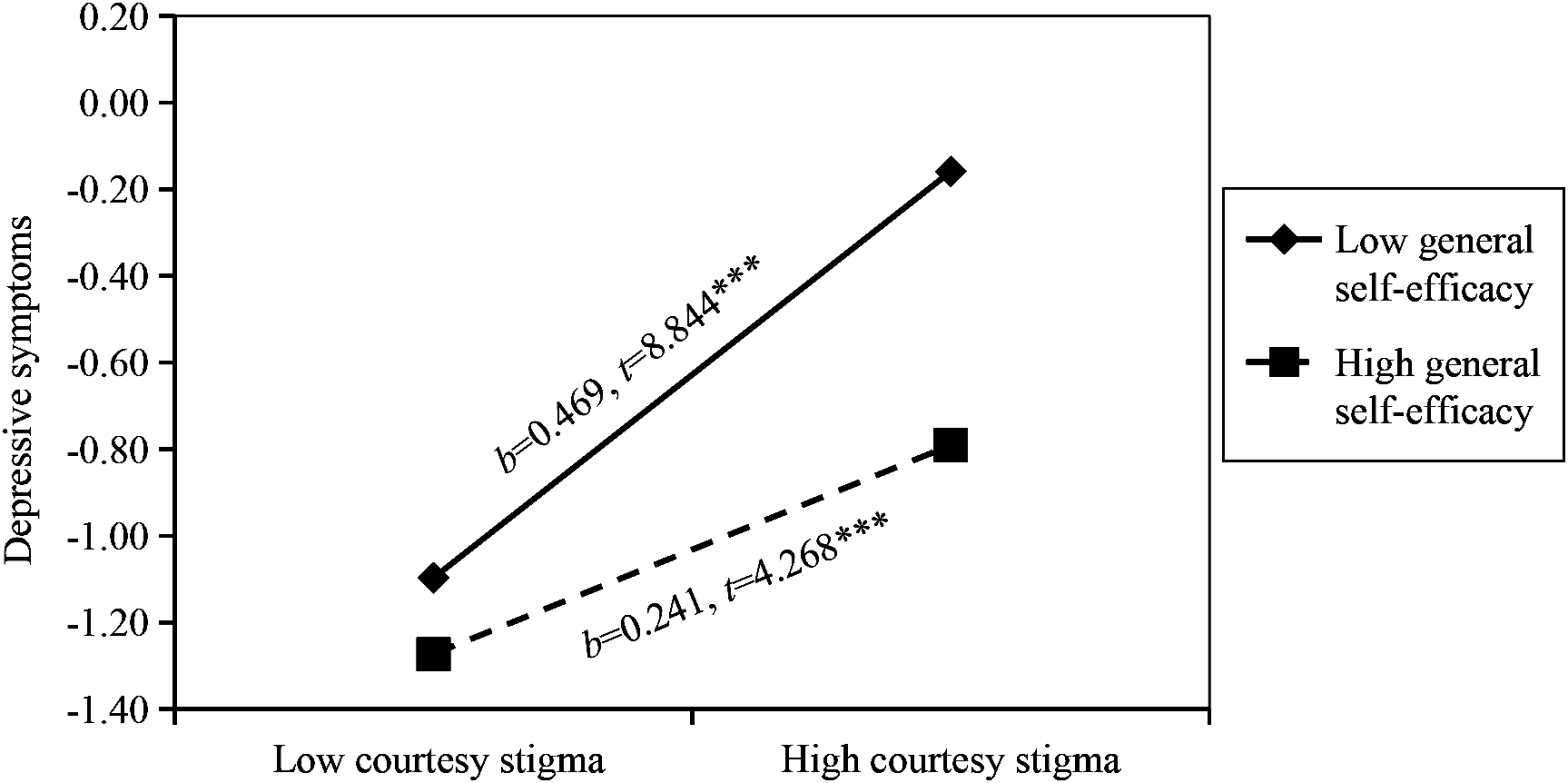
Moderating effect of general self-efficacy on the relationship between courtesy stigma and depressive symptoms. *b* = slope; *** *P*<0.001.

In the second step, adding courtesy stigma and general self-efficacy, both anxiety symptoms (*F* = 14.478, *R* ^2^ = 0.305, Δ*R* ^2^ = 0.209, *P* < 0.001) and depressive symptoms (*F* = 15.335, *R* ^2^ = 0.317, Δ*R* ^2^ = 0.208, *P* < 0.001) model fit were improved. Courtesy stigma showed significant main effects on both anxiety symptoms (*B* = 0.374, *P* < 0.001) and depressive symptoms (*B* = 0.366, *P* < 0.001), which supported hypothesis 1. General self-efficacy also showed significant main effects on both anxiety symptoms (*B* = -0.200, *P* < 0.001) and depressive symptoms (*B* = -0.210, *P* < 0.001), which supported hypothesis 2. In the anxiety symptoms model, the courtesy stigma × general self-efficacy interaction term significantly explained an additional 1.9% of the variance in the third step (*F* = 14.533, *R* ^2^ = 0.324, Δ*R* ^2^ = 0.019, *P* < 0.001). Interaction was significant negative correlation with anxiety symptoms (*B* = -0.106, *P* < 0.01), suggested that general self-efficacy played a moderating role between courtesy stigma and anxiety symptoms. We followed Aiken and West’s procedures and plotted the relationship under high (1 SD above the mean) and low (1 SD below the mean) levels of general self-efficacy. Simple slope analysis exhibited that the impact of courtesy stigma on anxiety symptoms was different at high (*b* = 0.258, standard error (SE) = 0.056, *t* = 4.567, *P* < 0.001) and low (*b* = 0.470, SE = 0.053, *t* = 8.870, *P* < 0.001) levels of general self-efficacy. In other words, when general self-efficacy was lower, the relationship between courtesy stigma and anxiety symptoms became stronger (**Figure 2**). In the depressive symptoms model, the courtesy stigma × general self-efficacy interaction term significantly explained an additional 2.2% of the variance in the third step (*F* = 15.573, *R* ^2^ = 0.339, Δ*R* ^2^ = 0.022, *P* < 0.001). Interaction was significant negative correlation with depressive symptoms (*B* = -0.114, *P* < 0.001), suggested that general self-efficacy played a moderating role between courtesy stigma and depressive symptoms. Simple slope analysis exhibited that the impact of courtesy stigma on depressive symptoms was different at high (*b* = 0.241, SE = 0.056, *t* = 4.268, *P* < 0.001) and low (*b* = 0.469, SE = 0.053, *t* = 8.844, *P* < 0.001) levels of general self-efficacy. In other words, when general self-efficacy was lower, the relationship between courtesy stigma and depressive symptoms became stronger (**Figure 3**). This supports hypothesis 3.

## Discussion

Anxiety and depressive symptoms are prevalent in parents of children with ASD, yet they often go ignored (51). This study explored the association between courtesy stigma, general self-efficacy and anxiety and depressive symptoms in parents of children with ASD in eastern China. We believed that our research findings contribute to the expansion of previous perspectives, while also providing insights for psychological intervention aimed at parents of children with ASD. Our study also offered a theoretical basis for improving the effectiveness of rehabilitation therapy for children with ASD. Our results supported all research hypotheses, indicating a positive correlation between courtesy stigma and anxiety and depressive symptoms, a negative correlation between general self-efficacy and anxiety and depressive symptoms, and that general self-efficacy could moderate the relationship between courtesy stigma and anxiety and depressive symptoms.

In this study, we found a significant correlation between the severity of children with ASD and parental anxiety and depressive symptoms. This was consistent with previous research indicating that parents of severely affected children with ASD were more likely to experience anxiety and depressive symptoms compared with parents of mildly affected children with ASD (8, 52). Previous research also revealed that as behavioral problems increased in children with ASD, parental acceptance decreased, leading to a significant increase in parenting mental health issues (9, 53). This may be attributed to the fact that children with ASD with more severe conditions required more attention and special care, while their additional behavioral problems and communication difficulties presented heightened challenges for parents. Notably, ASD severity is associated with increased parenting stress experienced by caregivers, which further increases the likelihood of unemployment (9). Our findings also suggested that unemployment status serve as one of the risk factors contributing to parental anxiety and depressive symptoms. Unemployment status could impose a substantial financial burden on families with children diagnosed with ASD, thereby significantly contributing to parental anxiety and depressive symptoms (54, 55). Consequently, the focus of psychological intervention for parents of children with ASD should be on those with severe ASD and unemployed parents.

Previous findings indicated that parents of children with ASD could experience public and courtesy stigma, which heightened parental affective symptoms, further impacting their mental well-being (17, 56). Consistent with previous research, our study suggested that the courtesy stigma was a significant associated factor to anxiety and depressive symptoms in parents of children with ASD (21). On the one hand, this may be attributed to the erroneous accusations though hereditary transmission and suboptimal parenting practices leading to disability in children. On the other hand, parents may be blamed due to the public’s misconceptions of their children’s social defects and behavioral issues (30, 53, 57). Moreover, in the face of public opposition and discrimination, parents of children with ASD worried that the courtesy stigma could have a detrimental impact on their children’s life opportunities and future development (58). These can cause mental health problems for parents. Given the harm and impact that courtesy stigma poses to parents, it was essential to implement appropriate intervention measures. For practitioners, additional interventions focused on trait mindfulness and social support may be beneficial in mitigating the impact of courtesy stigma (20, 28). Timely intervention can not only help parents of children with ASD in developing resistance and resilience against stigma, but also protect themselves from adverse psychological outcomes and enhance the rehabilitation of their children with ASD (20).

General self-efficacy is associated with psychological well-being, and strengthening parents’ general self-efficacy may become an indispensable component of rehabilitation therapy for children with ASD (59). Our research suggested that general self-efficacy serves as a protective factor against anxiety and depressive symptoms in parents of children with ASD. More importantly, our research indicated that general self-efficacy in parents of children with ASD could moderate the relationship between courtesy stigma and anxiety and depressive symptoms. In general, enhancing general self-efficacy in parents of children with ASD could mitigate the adverse impact of courtesy stigma on anxiety and depressive symptoms. Specifically, the association between courtesy stigma and anxiety and depressive symptoms was exacerbated at low level of general self-efficacy, while it was buffered at high level of general self-efficacy. This finding aligned with previous research conducted on caregivers of children with physical disabilities and patients with epilepsy (60, 61). People with solid general self-efficacy tend to have good emotional regulation ability (40). Conversely, parents of children with ASD who have low general self-efficacy frequently experience heightened negative emotions and psychological stress, which can hinder their motivation and decision-making when confronted with challenges or stress (62). They tend to exhibit adverse mental health conditions such as frustration, nervous, withdrawal, worry, fear, anxiety, and depression when encountering more challenges (63, 64). However, a higher level of general self-efficacy could serve as a stable internal resource for parents of children with ASD to cope with stress and challenges, thereby mitigating the negative impact of courtesy stigma on their mental health. Our findings clearly demonstrated that enhancing the general self-efficacy of parents was crucial in alleviating the adverse effect of courtesy stigma on anxiety and depressive symptoms. Therefore, psychological intervention targeting parents of children with ASD should incorporate specific strategies aimed at improving general self-efficacy. Previous studies have shown that increasing mental health education and regular exercise (such as bodybuilding, football, dance) can improve general self-efficacy (65, 66).

### Limitations

There are several limitations in this study that need explanation. Firstly, it was a cross-sectional study, which restricted the possibility of causal inference between research variables. Longitudinal studies are needed to confirm and evaluate these findings in the future. Secondly, all participants in this study were from Lianyungang, Jiangsu Province, Eastern China. Therefore, the results may only represent regions with similar socio-cultural context and economic condition. Thirdly, the majority of parents of children with ASD recruited for this study were primarily recruited from local medical healthcare and rehabilitation facilities. These parents may have access to more medical expertise knowledge and peer support. It might be necessary to conduct research on population outside healthcare institutions (including communities and households) when considering future studies. Finally, measurement of variables such as anxiety or depressive symptoms in this study was based solely on self-assessment, which may introduce bias. Future investigations might benefit from employing diverse methodologies for data collection.

## Conclusion

In summary, there was a positive correlation between the courtesy stigma and anxiety and depressive symptoms in parents of children with ASD. General self-efficacy has a negative correlation with anxiety and depressive symptoms. General self-efficacy could moderate the effects of courtesy stigma on anxiety and depressive symptoms. These findings emphasized the practical significance of enhancing general self-efficacy in parents of children with ASD. Therefore, in addition to reducing the courtesy stigma, strengthening general self-efficacy as a strategy to alleviate the impact of courtesy stigma on anxiety and depressive symptoms can also help improve the psychological well-being in parents of children with ASD, and ultimately mitigate the detrimental consequence arising from anxiety and depressive symptoms.

## Data Availability

The original contributions presented in the study are included in the article/supplementary material. Further inquiries can be directed to the corresponding authors.
